# Improvement Effect of Mitotherapy on the Cognitive Ability of Alzheimer’s Disease through NAD^+^/SIRT1-Mediated Autophagy

**DOI:** 10.3390/antiox12112006

**Published:** 2023-11-16

**Authors:** Xiaoxi Yang, Peiyu Zhou, Zizhen Zhao, Jingli Li, Zhigang Fan, Xiaorong Li, Zhihong Cui, Ailing Fu

**Affiliations:** School of Pharmaceutical Sciences, Southwest University, Chongqing 400715, China; yxx895623@email.swu.edu.cn (X.Y.); zpy0408@email.swu.edu.cn (P.Z.); zhaozizhen0512@foxmail.com (Z.Z.); swuljl990822@email.swu.edu.cn (J.L.); fanzg@swu.edu.cn (Z.F.); lixrong@swu.edu.cn (X.L.); cuizhihong@swu.edu.cn (Z.C.)

**Keywords:** AD, mitotherapy, autophagy, NAD^+^/SIRT1

## Abstract

To date, Alzheimer’s disease (AD) has grown to be a predominant health challenge that disturbs the elderly population. Studies have shown that mitochondrial dysfunction is one of the most significant features of AD. Transplantation therapy of healthy mitochondria (mitotherapy), as a novel therapeutic strategy to restore mitochondrial function, is proposed to treat the mitochondria−associated disease. Also, the molecular mechanism of mitotherapy remains unclear. Here, we applied the mitotherapy in AD model mice induced by amyloid−β (Aβ) plaque deposition and suggested that autophagy would be an important mechanism of the mitotherapy. After the healthy mitochondria entered the defective neuronal cells damaged by the misfolded Aβ protein, autophagy was activated through the NAD^+^−dependent deacetylase sirtuin 1 (SIRT1) signal. The damaged mitochondria and Aβ protein were eliminated by autophagy, which could also decrease the content of radical oxygen species (ROS). Moreover, the levels of brain−derived neurotrophic factor (BDNF) and extracellular−regulated protein kinases (ERK) phosphorylation increased after mitotherapy, which would be beneficial to repair neuronal function. As a result, the cognitive ability of AD animals was ameliorated in a water maze test after the healthy mitochondria were administrated to the mice. The study indicated that mitotherapy would be an effective approach to AD treatment through the mechanism of autophagy activation.

## 1. Introduction

At present, Alzheimer’s disease (AD) has come to be a severe challenge in global brain health. The prevalence and mortality rate of AD patients are rising rapidly with the increase in the elderly population [1,2,3,4,5]. Progressive cognitive impairment is one of the main symptoms of AD patients. In recent years, many potential reagents to improve AD symptoms have been studied, but about 99.6% of them fail in clinical trials [6,7,8,9,10]. Therefore, it is urgent to develop new effective approaches to treat AD.

Several hypotheses have been proposed to explain the causes of neuronal injury in AD brains, which include metabolic disorders, misfolded Aβ deposition, excessive phosphorylation of the Tau protein, etc. Metabolic disorder is the earliest event in AD progress, in which mitochondrial dysfunction is primarily responsible for it, since mitochondria are vital to the neuronal metabolism [11,12,13]. During the aging process, mitochondrial damage, including mtDNA mutation, the reduction of mitochondrial enzyme activities, and mitochondrial membrane potential, will gradually accumulate [14]. When the damage exceeds the threshold, neurons will show significant metabolic disorders, which is the earliest occurrence of AD. Subsequently, a series of metabolic disorders occur in cells due to insufficient energy, such as autophagy, Aβ protein misfolding, etc. These events in turn exacerbate the mitochondrial damage by a vicious cycle, thereby leading to the continuous progression and deterioration of AD [15]. In AD patients and animals, a large number of damaged mitochondria are observed under electron microscopy [16]. Enzyme activities of the electron transport chain (ETC) and tricarboxylic acid (TCA) cycle are decreased in neuronal mitochondria, such as α−ketoglutarate dehydrogenase (α−KGDH), pyruvate dehydrogenase (PDH), and the ETC complex I [12,17]. The decreased oxidative phosphorylation ability of neuronal mitochondria leads to a decrease in ATP production and the destruction of redox balance, which affects the normal function of cells. Moreover, mitochondrial autophagy dysfunction is usually the cause of the pathological deterioration of AD, among which mitochondrial dysfunction plays a central role in the pathogenesis. ATP reduction of insufficient cell energy supply will also inhibit the energy-consuming mitochondrial autophagy process. According to the mitochondrial hypothesis of AD, defective mitochondria will lead to increased reactive oxygen species (ROS), Aβ deposition, suppressed autophagy, and other pathological features related to AD [18,19,20]. Thus, recovery of mitochondrial function to elevate energy generation and activate autophagy is considered a potential approach to prevent AD progression. 

In recent years, a novel therapeutic strategy called transplantation therapy of healthy mitochondria (mitotherapy) has attracted widespread attention for treating diseases related to mitochondrial dysfunction. Mitotherapy is a bioengineering technology of organelle transplantation that involves the transfer of healthy mitochondria to defective cells, where healthy mitochondria replace damaged mitochondria to restore the metabolism and signal transduction, and studies have suggested that exogenous mitochondria can directly transform into cultured xenogeneic cells and improve their mitochondrial function [21,22]. In clinical trials, the therapeutic strategy was used to treat patients with heart failure caused by ischemia/reperfusion injury, and most patients showed significant improvement in heart function and successfully detached from extracorporeal membrane oxygenation [23]. In another clinical study, patients who received mitochondria had faster cardiac function recovery and lower incidences of cardiovascular adverse events than those without mitochondrial transplantation [24]. Moreover, the effectiveness and safety of mitochondrial augmentation therapy have been validated in improving patients’ single large-scale mitochondrial DNA (mtDNA) deletion syndromes [25].

Nevertheless, the molecular mechanism of mitotherapy remains unclear. In this study, mitotherapy was used to alleviate the cognitive impairment of AD, and the results revealed that autophagy activated by NAD^+^/SIRT1 played an important role in recovery cell function, besides increasing the energy supply of the mitochondria. The study for the first time suggests that NAD^+^/SIRT1−mediated autophagy would be one of the main mechanisms of mitotherapy.

## 2. Materials and Methods

### 2.1. Animals

Healthy male C57BL/6 mice (weight 25~30 g) were obtained from Hunan SJA Animal Co., Ltd. (Hunan, China). The mice were supplied with drinking water and food freely in a standard SPF animal laboratory. All animal experiments were approved by the Institutional Animal Care and Use Committee of Southwestern University.

### 2.2. Mitochondrial Isolation

Mitochondria were isolated from mouse brain tissue by differential centrifugation according to the report [26]. The operation was performed at 0~4 °C. Briefly, mouse brain tissue was homogenized with an SE buffer solution (30 mM Tris, 10 mM EDTA−2Na, 2.5 mM CaCl_2_, 250 mM sucrose) and centrifuged at 3000 rpm. Then, the supernatant was centrifuged at 12,000 rpm for 15 min, and the precipitation was resuspended in the buffer solution. Mitochondria were counted under an optical microscope (Chongqing Optec Instrument Co., Ltd., Chongqing, China) after Janus Green B staining. The mitochondrial concentration was determined with the bicinchoninic acid (BCA) assay. One mg/mL mitochondrial solution was counted to contain 1.5 × 10^7^ mitochondria. Mitochondria were labeled using MitoTracker Red CMXRos (Invitrogen Co., Carlsbad, CA, USA) by the manufacturer’s protocol and observed. 

### 2.3. Mitochondrial Membrane Potential (MMP) Measurement

MMP was measured to assess the integrity of the isolated mitochondria. JC−1 is a kind of cationic lipid fluorescent dye that can access mitochondria to form an aggregate with a high MMP and emits a red fluorescence (excitation wavelength 490 nm, emission wavelength 525 nm). When the MMP reduces, JC−1 is released from mitochondria in monomer form and emits a green fluorescence (excitation wavelength 525 nm, emission wavelength 590 nm). The fluorescence intensity was detected by a fluorescence spectrometer (BioTek Instrument Co., Winooski, VT, USA) after the mitochondria were incubated with the JC−1 staining solution at 37 °C for 15 min. Carbonyl cyanide m−chlorophenylhydrazone (CCCP) that can induce MMP loss was used as a positive control. 

### 2.4. Cell Culture

Human neuroblastoma SH-SY5Y cells were cultured in DMEM high−glucose media containing 10% fetal bovine serum, 100 U/mL penicillin, and 100 μg/mL streptomycin at 37 °C in a cell incubator with 5% CO_2_. All cell−culture materials were purchased from Beijing Labgic Co., Ltd., Beijing, China. 

### 2.5. Preparation of Aβ_1−42_−Damaged Cells and Cell Viability Measurement

Aβ_1−42_ was synthesized from Shanghai GenicBio Biotech. Co., Ltd., Shanghai, China. After 10 μM Aβ_1−42_ was added to the SH−SY5Y cell media for 60 h incubation, the isolated mitochondria were put into the media for further incubation [27]. The broken mitochondria with repeated freeze–thaw cycles were used as a negative control. The cell viability was measured by using Alamar Blue reagent by the manufacturer’s protocol (Beyotime Biotech. Co., Ltd., Shanghai, China). Cells not treated with mitochondria were utilized as the blank. The relative cell vitality was calculated by the formula: cell viability (%) = OD (sample − blank)/OD (control − blank) × 100%.

### 2.6. Biochemical Assay

The redox−related indexes, including ROS, malondialdehyde (MDA), reduced glutathione (GSH), oxidized glutathione (GSSG), total antioxidant capability (T−AOC), glutathione peroxidase (GSH−Px), and superoxide dismutase (SOD), respectively, were measured by the commercial kits (Beijing Suolaibao Co., Ltd., Beijing, China). The ROS content was determined by DCFH-DA [28]. The assay of MDA involves the reaction of MDA with thiobarbituric acid (TBA), which leads to the formation of MDA−TBA_2_ adducts (maximum absorption 535 nm) [29]. GSH, GSSG, and GSH-Px were assayed based on the principle that GSH can interact with 5,5′−dithiobis−2−nitrobenoic acid to form 2−nitro−5−thiobbenzoic acid (maximum absorption 412 nm) [30,31]. The T−AOC detection is based on the reaction of antioxidant substances reducing ferric−tripyridyltriazine (Fe^3+^−TPTZ) to blue Fe^2+^−TPTZ (maximum absorption 593 nm) in an acidic environment [32]. And, the principle of SOD detection is that SOD can inhibit the formation of blue formazan from the O^2-^−reducible nitrogen blue tetrazole (maximum absorption 560 nm) [33]. The contents of NAD^+^, NADH, ADP, and ATP were detected by HPLC (Shimadzu Co., Kyoto, Japan) [34]. Moreover, the activities of mitochondrial TCA−cycle−related enzymes, including PDH, isocitrate dehydrogenase (ICDHm), α−KGDH, succinate dehydrogenase (SDH), as well as mitochondrial ETC complexes I, II, III, and IV, respectively, were detected according to the operation manuals (Beijing Suolaibao Co., Ltd., Beijing, China). The detection of PDH according to PDH catalyzes pyruvate dehydrogenation along with the reduction of 2,6−dichloroindophenol, which results in decreased absorption at 605 nm [35]. The detection principle of ICDHm involves isocitrate dehydrogenase catalyzing isocitrate to produce α-ketoglutaric acid (maximum absorption 505 nm) [36]. The assay of α−KGDH, according to α−KGDH catalyzing NAD^+^ to NADH, has a characteristic absorption at 340 nm [37]. SDH detection is based on the principle that SDH can catalyze the dehydrogenation of succinic acid to reduce 2,6−dichloroindophenol, resulting in a change in absorbance at 600 nm [38]. And, the ETC complexes I, II, III, and IV were detected by an enzyme-linked immunosorbent assay [39].

### 2.7. Immunofluorescence Staining

The cells were fixed with 4% paraformaldehyde and permeated with 0.1% Triton−X100, then blocked in 2% bovine serum albumin (BSA) for 60 min. The cells were respectively incubated at 4 °C overnight with the primary antibody of rabbit anti−BDNF and rabbit anti−Aβ_1−42_ (Beyotime Biotech. Co., Ltd., Shanghai, China). Subsequently, FITC−labeled goat anti−rabbit IgG (Wuhan Servicebio Co., Ltd., Wuhan, China) was added and incubated for 60 min. Afterward, cell nuclei were labeled by the Hoechst 33342 (Beyotime Biotech. Co., Ltd., Shanghai, China) and observed. 

### 2.8. Western Blot

The cells were lysed with the aid of the RIPA buffer. After centrifugation at 10,000 rpm for 5 min, the BCA assay was used to determine the protein concentration of the supernatant. Protein samples were dissociated by SDS−PAGE and then transferred to polyvinylidene fluoride membranes under an ice bath. Subsequently, the membrane was incubated with a blocking solution (5% skim milk dissolved in TBST buffer). The primary antibodies (anti−FOXO3, anti−BNIP3, anti−LC3A/B, anti−ERK1/2, anti−β−actin, anti−BDNF, and anti−p−ERK1/2) were respectively used to detect the corresponding proteins. Afterward, the membranes were placed in the secondary antibody solution (HRP−labeled goat anti−rabbit IgG). Finally, the ECL imaging system (Shanghai Clinx Science Instruments Co., Ltd., Shanghai, China) was used to develop and take photographs.

### 2.9. Cell Autophagy Detection

The cell autophagy was measured by Western blot, transmission electron microscopy (TEM), and monodansylcadaverine (MDC) staining, respectively. For MDC staining, MDC staining solution was added into the cell−culture wells at 37 °C for 30 min incubation, and then the fluorescence microscope (Optec Instrument Co., Ltd., Chongqing, China) was used to observe the cells. For the TEM observation, the cells were fixed with 2.5% glutaraldehyde for 24 h at 4 °C, and then TEM (Hitachi Co., Tokyo, Japan) was used to observe the mitochondria and autophagy. 

### 2.10. Transcriptomic Analysis

Healthy mitochondria (1.5 × 10^6^/mL) were added into Aβ_1−42_−damaged cells for 4 h; then, the cells were gathered for transcriptomic analysis. Total mRNA was extracted and enriched by Oligo(dT) magnetic beads. After the mRNA was fragmented into 300 bp, cDNA was synthesized by RT-PCR. The gene−expression level of each transcript was detected, and differential expression genes (DEGs) between Aβ_1−42_−damaged cells and mitochondria−treated cells were identified following the common criteria of |log2FoldChange| > 1 and the *p*−value < 0.05. Functional enhancement of GO, as well as KEGG pathway analysis, were carried out to assess the DEG function. 

### 2.11. Group Assignment and Morris Water Maze

Twenty−seven mice were assigned into three groups randomly with nine mice in each group. AD model mice were constructed by the microinjection of 200 μmol/L Aβ_1−42_ on both sides of the hippocampus in the model group and the mitotherapy group, and the sham group was administrated an equal dose of saline. Following 3 days’ injection, each mouse in the mitotherapy group was injected with mitochondria at a dose of 3 × 10^6^/0.2 mL through the caudal vein. Meanwhile, each mouse in the sham group and model group was respectively injected with an equal dose of saline. All groups received the injection continuously for 4 days.

The Morris water maze (Huaibei Zhenghua Instruments Co., Ltd., Huaibei, China) test was implemented to assess learning, cognitive, and memory functions [40]. The equipment contains a circular pool, and the water temperature is kept at 23 ± 2 °C. The pool is equally set to 4 quadrants. An invisible escape platform (10 cm) was placed 1 cm underwater. A camera−connected real-time tracking system was used for record and data analysis. In the navigation experiment, mice were respectively tested in each quadrant in the morning and afternoon per day for 3 days. The time when the mouse climbed onto the platform was used as the escaping time. For mice that could not seek out the platform within 180 s, the escaping time was set as 180 s. The spatial probe test that the platform moved away was conducted 24 h following the navigation test. Swimming traces and the time that mice lingered in the target quadrant were recorded within 60 s. 

### 2.12. Thioflavin−T Staining of Aβ

Mouse brain tissues were fixed in paraformaldehyde for 24 h, and then 10%, 20%, and 30% sucrose solution dehydration step-by-step. Subsequently, sections (30 μm thickness) were cut by microtome cryostat. Tissue sections were stained with 0.3% thioflavin-T and observed. 

### 2.13. Data Statistics

Statistical analysis was implemented using GraphPad Prism 9, and the results were manifested as mean ± SD. Distinction between the two and multiple groups were compared using the *T*−test and one−way ANOVA, respectively. Significant and highly significant differences were *p* < 0.05 and *p* < 0.01, respectively. 

## 3. Results

### 3.1. The Mitochondria Increased the MMP of Aβ-Damaged SH−SY5Y Cells

**Figure 1:**
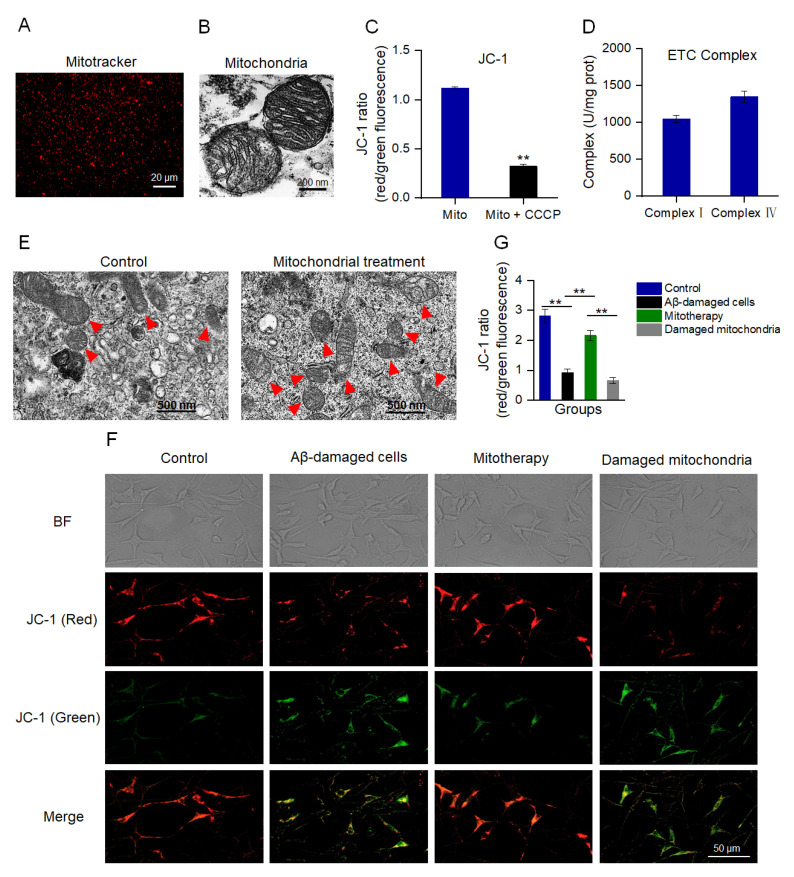
Isolated mitochondria entered the cells and increased the MMP of Aβ−damaged cells. (**A**) The isolated mitochondria were stained by MitoTracker Red CMXRos. (**B**) TEM observation of mitochondrial structure. (**C**) MMP detection of the isolated mitochondria by JC−1. Mito, mitochondria. Mito + CCCP, CCCP pretreated before mitochondrial addition. Each experiment was carried out independently 6 times (n = 6). ** *p* < 0.01 compared with the mitochondria without CCCP treatment. (**D**) Activities of mitochondrial ETC complexes I and IV. (**E**) TEM observation of mitochondria in cells. Red arrows pointed to mitochondria. (**F**) Healthy mitochondria entered the Aβ−damaged cells and enhanced the MMP. The broken mitochondria that underwent three consecutive freeze−thaw cycles were used as the negative control. (**G**) JC−1 ratio after mitochondrial treatment. Each experiment was performed independently 6 times (n = 6). ** *p* < 0.01.

MMP is an important indicator to denote mitochondrial integrity and the ability of oxidative phosphorylation (OXPHOS) [41,42]. MitoTracker Red CMXRos is a red fluorescent dye that can specifically label mitochondria with a normal MMP (Figure 1A). Also, TEM was implemented to assess the structural integrity of the isolated mitochondria. The images exhibited that the mitochondrial inner and outer membranes were visible, and the cristae arrangement was compact and regular (Figure 1B). The JC−1 staining showed that the isolated mitochondria had a high ratio of aggregate to monomers (red/green fluorescence), while the ratio decreased significantly after the CCCP pretreatment (Figure 1C), suggesting the isolated mitochondria possessed a high MMP. Moreover, the activities of both complex I and IV were higher than 1000 U/mg prot (Figure 1D), which were the normal values of healthy mitochondria that were consistent with the previous report [43]. These results manifested that the structure of the isolated mitochondria was intact, which could be applied for mitotherapy. 

To evaluate whether the isolated mitochondria could enter the SH−SY5Y cells, the cells were observed by TEM at 10 min after mitochondrial addition into the cell media. Under the TEM, the number of intracellular mitochondria elevated and the morphology was intact (Figure 1E). Further, the MMP was measured to identify whether the isolated mitochondria would enter Aβ−damaged cells and recover the membrane potential. As shown in Figure 1F,G, Aβ−damaged SH−SY5Y cells exhibited weak red fluorescence and strong green fluorescence compared with those of the normal cells, suggesting that the cells were impaired by the MMP decrease by Aβ. However, red fluorescence became strong after the isolated mitochondrial addition. Contrary to the cells treated with healthy mitochondria, the Aβ−damaged cells that were treated with mitochondrial fragments still showed weak red fluorescence (Figure 1F,G), indicating that mitochondrial integrity is crucial for mitotherapy.

### 3.2. Healthy Mitochondria Reduced Aβ Aggregation and Increased Cell Viability

**Figure 2:**
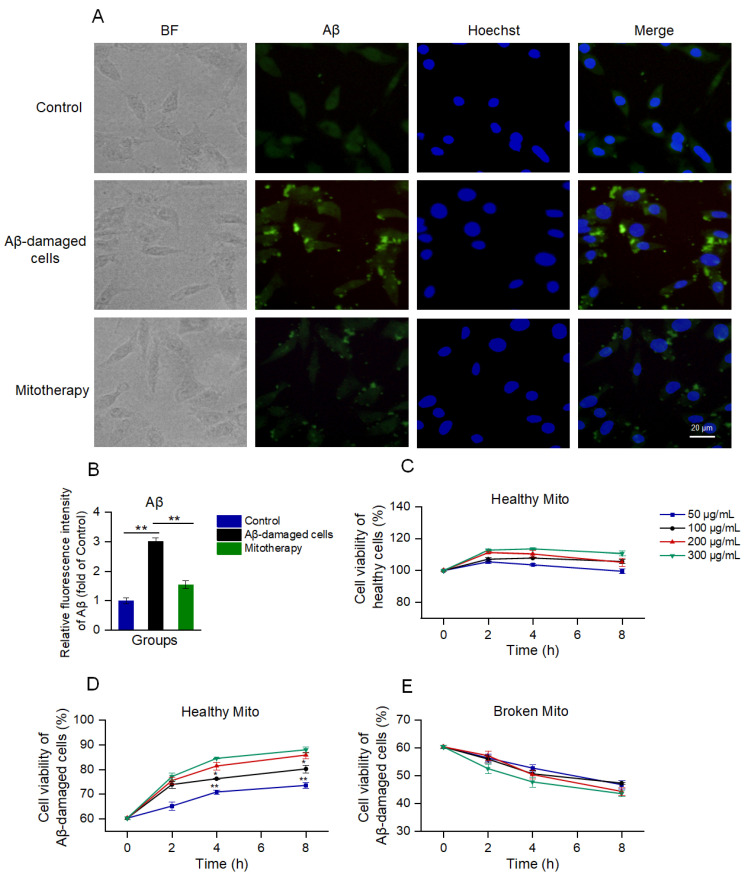
The healthy mitochondria could reduce Aβ content and increase cell viability. (**A**) Immunofluorescence staining of Aβ. (**B**) Fluorescence intensity of Aβ. ** *p* < 0.01. (**C**) Effect of healthy mitochondria on control cells. (**D**) Healthy mitochondria elevated the cell viability of Aβ−damaged SH-SY5Y cells. * *p* < 0.05, ** *p* < 0.01, vs. the 200 μg/mL group. (**E**) Broken mitochondria could destroy the cells. Six independent experiments were conducted in each group (n = 6).

Aβ deposition is a major pathological feature of AD. Here, the cell Aβ level was detected by immunofluorescence. The images exhibited that much of the aggregation that was labeled by the green fluorescence appeared after the Aβ treatment, whereas the Aβ deposition was significantly reduced following the mitochondrial addition (Figure 2A,B). 

Then, cell viability was detected by using Alamar blue to evaluate the effect of mitotherapy on cells. The results showed that the healthy mitochondria slightly increased the viability of normal cells within 2 h (Figure 2C); then, the viability gradually reduced. In the Aβ−damaged cells, the mitotherapy significantly enhanced the cell viability in a concentration− and time−dependent manner (Figure 2D). However, the broken mitochondria could cause significant decreases in cell viability, showing obvious cytotoxicity (Figure 2E). 

### 3.3. Mitotherapy Decreased Oxidative Stress and Enhanced the Energy Supply in Aβ−Damaged Cells

**Figure 3:**
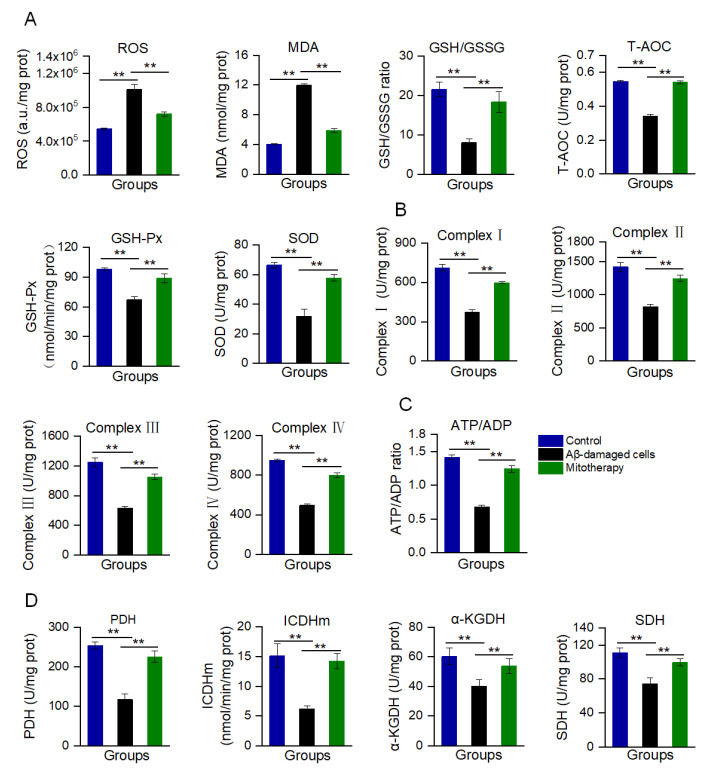
Biochemical measurement of oxidative stress and energy supply after mitotherapy. (**A**) Redox index assay. The levels of ROS, MDA, GSH/GSSG, T−AOC, and the activities of GSH−Px and SOD were detected at 8 h after mitochondrial addition into the cell media. (**B**) Activities of the mitochondrial ETC complexes I, II, III, and IV. (**C**) ATP/ADP ratio. (**D**) Activities of PDH, ICDHm, α-KGDH, and SDH. Six independent experiments were performed in each group (n = 6). ** *p* < 0.01.

Aβ accumulation and mitochondrial dysfunction can promote the production of excessive ROS. In the Aβ-treated cells, the ROS content was remarkably increased, as well as MDA, the end-production of lipid peroxidation (Figure 3A). Correspondingly, the antioxidant components, such as GSH, GSH−Px, SOD, and T−AOC, were decreased (Figure 3A). However, mitotherapy reversed the oxidative stress, resulting in an ROS content reduction and the elevation of antioxidant component levels. 

Moreover, mitochondrial function was determined through the measurement of ETC activity and ATP content, as well as the activities of key enzymes in the TCA cycle. The results are shown in Figure 3B−D. After the mitochondrial addition, all of the ETC activities significantly increased (Figure 3B), followed by the ATP/ADP ratio (Figure 3C). Meanwhile, the activities of PDH, ICDHm, α−KGDH, and SDH of the TCA cycle were significantly enhanced after the healthy mitochondrial treatment (Figure 3D). Collectedly, these results suggested that the mitotherapy could reduce the Aβ level and increase the ATP supply, which would rescue the neuronal function damaged by Aβ. 

### 3.4. Transcriptomic Analysis

**Figure 4:**
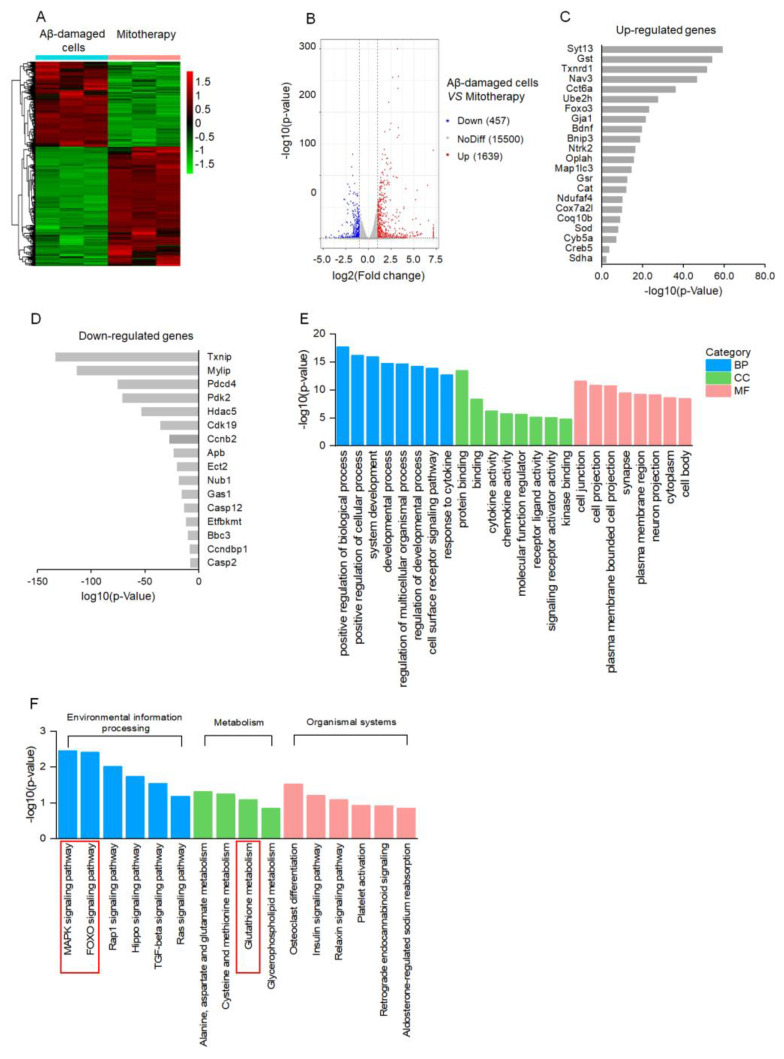
Transcriptomic analysis of mitotherapy in Aβ−damaged cells. (**A**) Heatmap of DEGs between mitochondria−treated and control cells. (**B**) Volcanic map of DEGs. (**C**) The most prominent 22 upregulated DEGs and (**D**) 16 downregulated DEGs after mitochondrial addition. (**E**) The dominant GO enrichment of the DEGs. (**F**) KEGG enrichment of the DEGs.

To clarify the molecular mechanism of mitotherapy on the cells, transcriptomic analysis was conducted after mitochondrial treatment for 4 h. The DEGs were identified based on the |log2FoldChange| > 1 and *p*−value < 0.05. A total of 2096 DEGs were regulated by the mitochondria, including 1639 upregulated genes and 457 downregulated genes (Figure 4A,B). Among the upregulated genes, DEGs related to neuronal repairment (*Syt13*, *Nav3*, *Gja1*, *Bdnf*, *Ntrk2*), antioxidant proteins (*Gst*, *Thnrd1*, *Gst*, *Cat*, *Sod*), autophagy (*Ube2h*, *FOXO3*, *Bnip3*, *Map1lc3*), and mitochondrial function (*Oplah*, *Ndufaf4*, *Cox7a2l*, *Coq10b*, *Cyb5a*, *Sdha*) were remarkably affected (Figure 4C). Among the downregulated DEGs, the transcription of the *Txnip* gene that is associated with oxidative stress was downregulated the most prominently, followed by cell−growth−inhibition genes *Mylip* and *Gas1*, as well as apoptosis−inducing genes *Pdcd4*, *Ect2*, *Casp12*, *Bbc3*, and *Casp2* (Figure 4D). Notably, the transcription of the Aβ gene, *Apb*, was significantly inhibited in the Aβ-damaged cells after mitotherapy.

According to the GO enrichment analysis, the 2096 DEGs were classified into three categories, in which the positive regulation of the biological process, cell junction, and protein binding, respectively, were the most prominent biological processes, molecular functions, and cell components, suggesting the recovery of cell function by healthy mitochondria (Figure 4E). Moreover, the MAPK and FOXO signal pathways, as well as the GSH metabolism, were involved in the mitotherapy after the KEGG enrichment (Figure 4F). 

### 3.5. The Healthy Mitochondria Induced Autophagy through the NAD^+^/SIRT1 Signal

**Figure 5:**
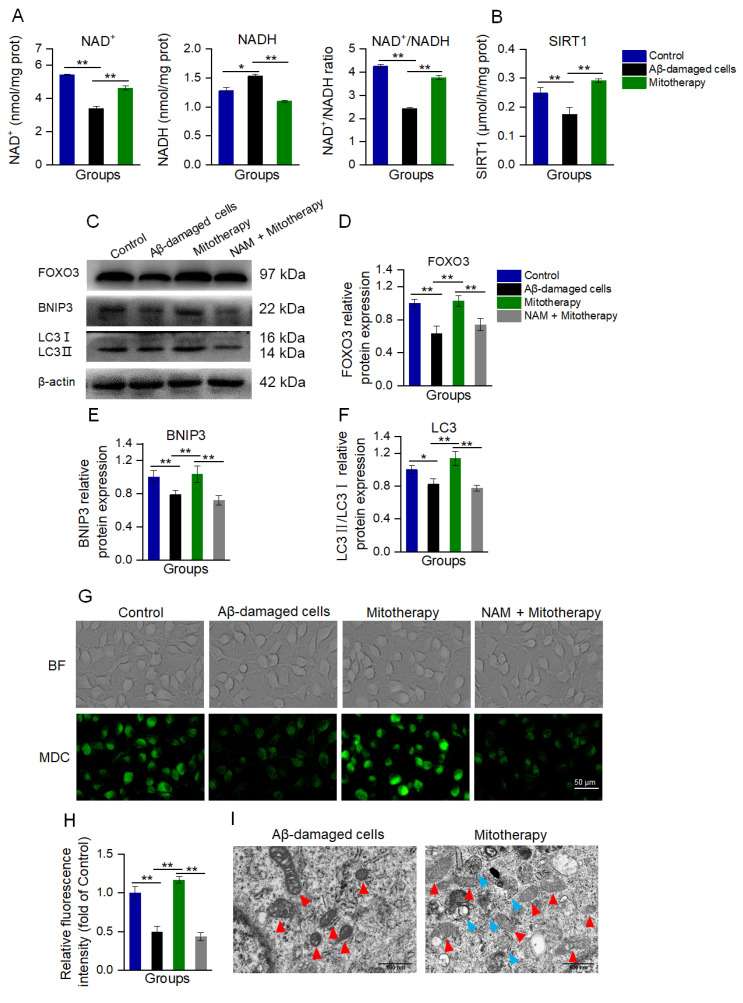
Mitotherapy activated autophagy in Aβ−damaged cells. (**A**) Contents of NAD^+^, NADH, and their ratios. (**B**) SIRT1 activity. (**C**) WB bands of autophagy−related proteins. (**D**) Gray values of FOXO3, (**E**) BNIP3, and (**F**) LC3, respectively. (**G**) MDC staining of autophagosome in cells. (**H**) Fluorescence intensity of MDC staining. (**I**) Autophagy and mitochondrial morphology by TEM observation. Red arrows pointed to mitochondria, and blue arrows to autophagosomes. Six independent experiments were performed in each group (n = 6). * *p* < 0.05, ** *p* < 0.01.

Since transcriptomic analysis suggested that the transcription of genes that were related to neuronal repair, antioxidant proteins, autophagy, and mitochondrial function were upregulated, and the MAPK and FOXO pathways were affected by the mitochondria, a transcription regulatory protein in the mitochondria−nuclear retrograde signaling pathway, NAD^+^−dependent sirtuin 1 (SIRT1), was speculated to be involved in the mitochondrial treatment. Here, the levels of NAD^+^ and NADH, as well as SIRT1 activity, respectively, were determined. As expected, both the NAD^+^/NADH ratio and SIRT1 activity were significantly increased after mitotherapy (Figure 5A,B). 

NAD^+^/SIRT1 is a positive regulator of autophagy, and SIRT1 without NAD^+^ activation can cause the deficiency of autophagy [44,45,46]. NAD^+^/SIRT1 activates the deacetylation of FOXO3 (a transcription factor responsible for autophagy and oxidative stress) to promote its transcriptional activity, and then upregulate the expression of FOXO3 downstream proteins [47,48]. Thus, the levels of FOXO3 and its downstream autophagy-related proteins, BNIP3 and LC3, were detected by WB. 

The results showed that the protein expressions of FOXO3, BNIP3, and LC3II/LC3I were significantly downregulated in the Aβ-damaged cells, whereas these protein levels were reversed after the mitochondrial addition (Figure 5C−F). Nevertheless, when the Aβ-damaged cells were pretreated by the SIRT1 inhibitor nicotinamide (NAM), mitochondria could not increase the expressions, indicating that SIRT1 activation would be an important factor in autophagy-related protein expression induced by the mitochondria. 

Further, autophagy was determined by MDC staining and TEM observation after the mitochondria were added into the cell media for 12 h of incubation. In Aβ−damaged cells, the green fluorescence of MDC decreased compared with the normal cells (Figure 5G,H), suggesting that the cellular autophagy ability was impaired by Aβ, which was aligned with the previous report [20,49]. However, the cells treated with healthy mitochondria showed strong fluorescence (Figure 5G,H), and several autophagosomes under the TEM (Figure 5I). Moreover, the mitochondria in the Aβ-damaged cells exhibited vacuoles, but those in the mitochondria−treated cells showed relatively normal morphology (Figure 5I). These indicated that the exogenous mitochondria could promote cell autophagy to eliminate the defective mitochondria. In addition, NAM withdrew the autophagy effect of the mitotherapy, confirming that the mitochondria promoted cell autophagy by activating SIRT1.

### 3.6. Mitotherapy Promoted BDNF Generation and ERK Phosphorylation through the NAD^+^/SIRT1 Signal

**Figure 6:**
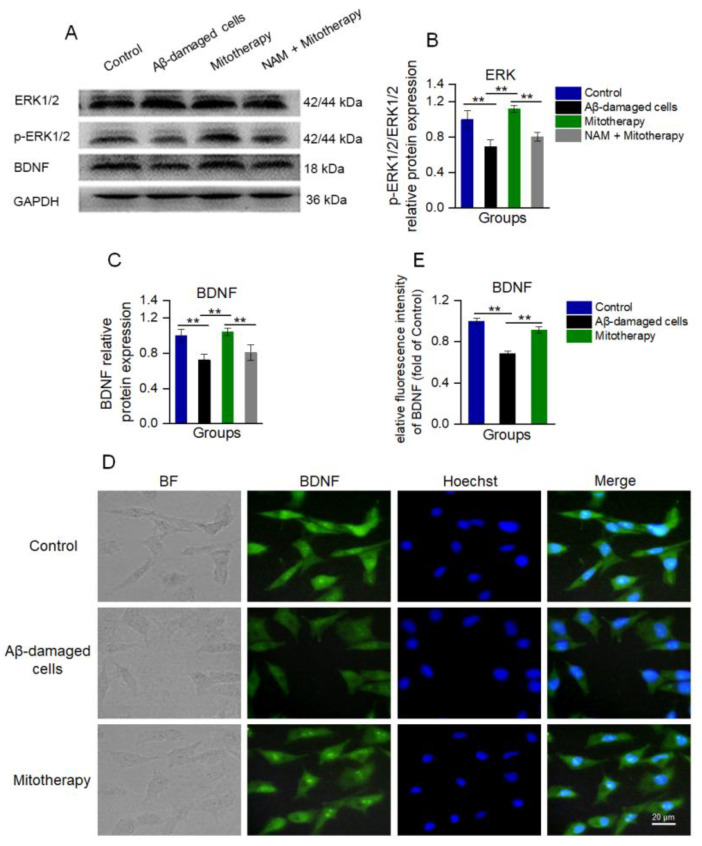
The healthy mitochondria increased the levels of BDNF production and ERK phosphorylation through NAD^+^/SIRT1. (**A**) WB bands of ERK, p−ERK, and BDNF. (**B**) The p−ERK/ERK ratio of the WB gray value. (**C**) Gray value of BDNF. (**D**) Immunofluorescence staining of BDNF in cells. (**E**) Immunofluorescence intensity of BDNF. Six independent experiments were performed in each group (n = 6). ** *p* < 0.01.

Since NAD^+^/SIRT1 can induce the protein expression of BDNF and facilitate ERK activation (phosphorylation), which are known to promote neurite growth and neuronal connectivity [50,51,52], WB was used here to detect the levels of BDNF, ERK, and phosphorylated ERK (p−ERK). The results showed that the levels of BDNF and p-ERK/ERK in the cells treated with mitochondria were significantly upregulated as compared to the untreated Aβ−damaged cells (Figure 6A−C), which were consistent with the transcriptomic analysis. Also, the immunofluorescence results of BDNF showed that the expression of BDNF was significantly heightened after mitotherapy (Figure 6D,E).

However, when the SIRT1 activity was blocked by its inhibitor NAM, the levels of BDNF and p−ERK/ERK in the cells of mitotherapy were reversed (Figure 6A−C), suggesting that SIRT1 activity would be an important upstream regulatory factor in mitotherapy. 

### 3.7. Mitotherapy Improved the Cognitive Ability of AD Mice

**Figure 7:**
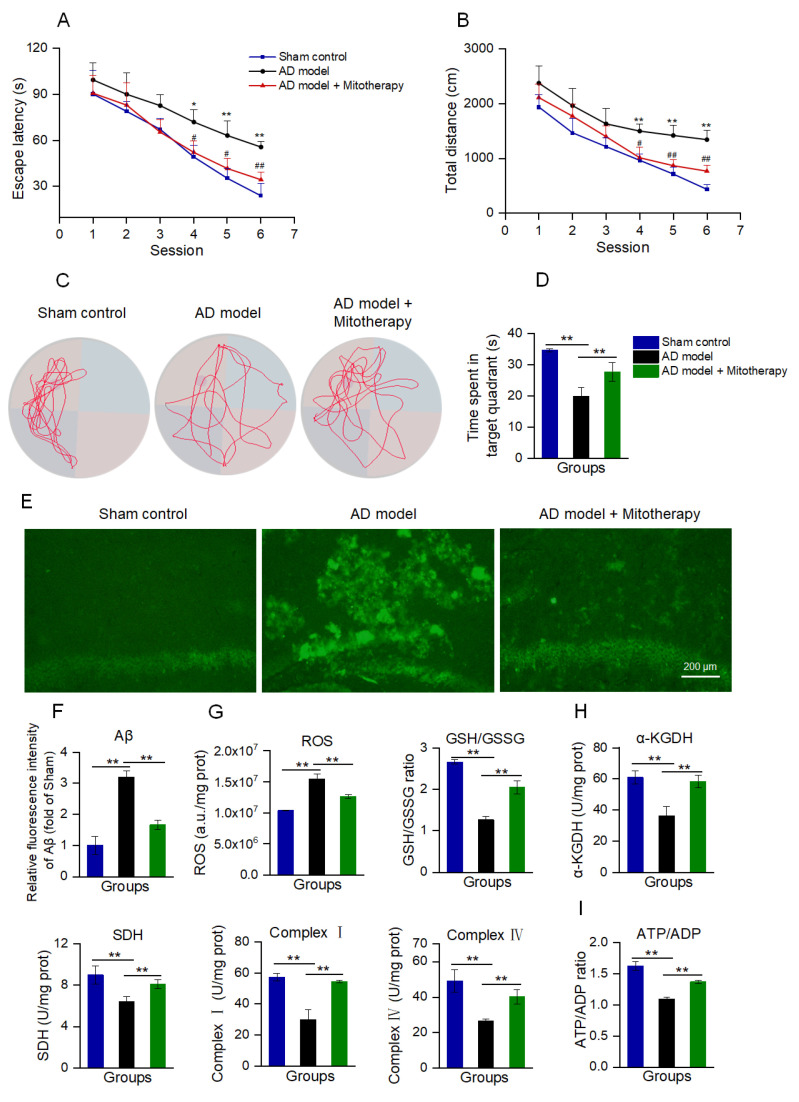
Mitotherapy improved the cognitive function of AD mice. (**A**) Escape latency in each group of mice in the Morris water maze test. Nine animals were used in each group (a total of twenty−seven animals). * *p* < 0.05, ** *p* < 0.01 vs. sham group; ^#^ *p* < 0.05, ^##^ *p* < 0.01, vs. AD model group. (**B**) Swimming distance in each group of mice. (**C**) Representative swimming routes of mice in the spatial probe test. (**D**) Time spent in the target quadrant in the probe test. (**E**) Aβ staining of mouse hippocampus by thioflavin−T. (**F**) Fluorescence intensity of Aβ stained by thioflavin−T. (**G**) ROS content and GSH/GSSG ratio. (**H**) Activities of α−KGDH, SDH, and ETC complexes I and IV. (**I**) ATP/ADP ratio. * *p* < 0.05, ** *p* < 0.01.

AD mice models were constructed by microinjecting Aβ_1−42_ into the bilateral hippocampus region of the brain according to the reports [53,54]. After 4 days of mitotherapy, the cognitive ability of mice was tested to evaluate the therapeutic effect of the healthy mitochondria. In the navigation test, both escape latency and swimming distance were prolonged in AD mice as compared with the sham control, while both of them became short after mitochondrial treatment (Figure 7A,B). In the spatial probe test, the time that the mitochondria-treated mice spent in the target quadrant significantly extended compared to the model mice (Figure 7C,D). The results suggested that mitotherapy could enhance the AD mice’s cognitive ability. 

To examine whether the mitochondria could reduce Aβ deposition in AD mice brains, Aβ was stained by thioflavin−T. The images showed that strong fluorescence of Aβ aggregation appeared in the hippocampal region of AD mice, whereas only weak fluorescence remained after mitotherapy (Figure 7E,F). 

Moreover, the results of the biochemical assay indicated that the mitotherapy reduced the ROS content in the AD mice’s hippocampus, and meanwhile increased the GSH/GSSG ratio, suggesting that the mitotherapy could decrease the oxidative stress caused by Aβ deposition (Figure 7G). Also, the enzyme activities in the TCA cycle, ETC, including α−KGDH, SDH, and complexes I and IV, were significantly increased after mitotherapy (Figure 7H), and correspondingly, the energy production enhanced and the ATP/ADP ratio increased (Figure 7I). 

### 3.8. Mitothreapy Regulated BDNF Production and Autophagy in the AD Mouse Brain

**Figure 8:**
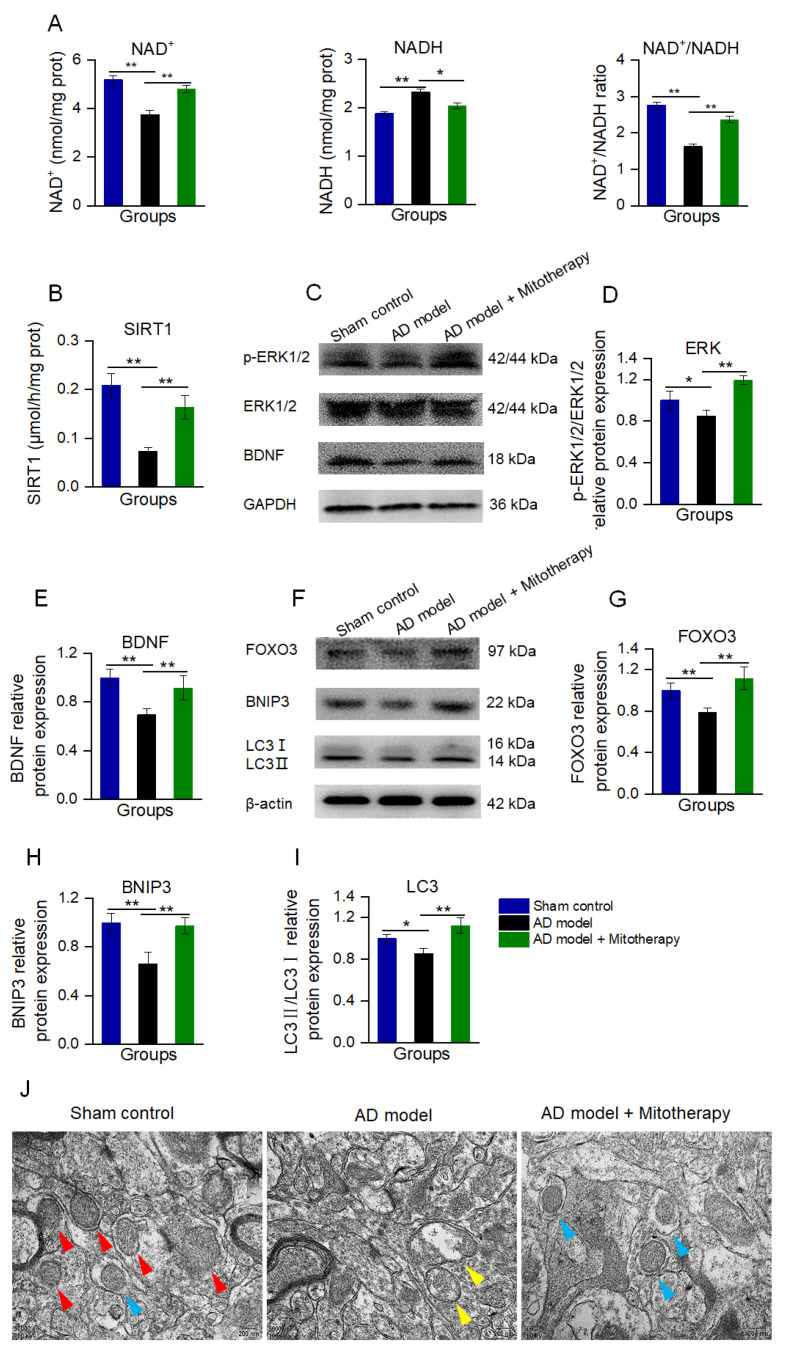
Mitotherapy induced BDNF production and autophagy in the hippocampus of the AD mouse brain. (**A**) The levels of NAD^+^, NADH, and the NAD^+^/NADH ratio. (**B**) SIRT1 activity. (**C**) WB of p−ERK, ERK, and BDNF. (**D**) Gray values of ERK and (**E**) BDNF. (**F**) WB of autophagy−related proteins. (**G**) Gray values of FOXO3, (**H**) BNIP3, and (**I**) LC3. (**J**) Autophagy in brain tissue under TEM. Red arrows pointed to healthy mitochondria, yellow arrows to damaged mitochondria, and blue arrows to autophagy. Nine independent experiments were performed in each group (n = 6). * *p* < 0.05, ** *p* < 0.01.

To confirm the mechanism of the mitotherapy, the levels of NAD^+^, NADH, and the activity of SIRT1 in the mouse hippocampus were determined. The results showed that the NAD^+^/NADH ratio and SIRT1 activity were significantly increased in AD mice after mitotherapy (Figure 8A,B), which is consistent with the effect of the mitochondrial treatment in the Aβ−damaged cells. Then, the protein expression of BDNF and the level of p−ERK/ERK of the hippocampus of the mice were measured by WB. As expected, the levels of BDNF expression and ERK phosphorylation in the AD mouse brain were remarkably upregulated in the mitochondria−treated mice (Figure 8C−E).

Moreover, the autophagy in the hippocampus of the mouse brain was detected by WB and TEM. As shown in Figure 8F−J, the levels of FOXO3, BNIP3, and LC3II/LC3I in AD mice were significantly reduced, whereas all of them were elevated after mitotherapy. In addition, defective mitochondria were observed under the TEM in the model mouse brain, but a number of autophagosomes appeared in the brain of mitochondria−treated mice (Figure 8J), suggesting that the defective mitochondria would be eliminated by the autophagosomes.

## 4. Discussion

AD is the most ordinary form of senile dementia, but there is still a lack of effective treatments to ameliorate the cognitive impairment. Mitochondrial injury is one of the most representative pathological features of AD. Energy deficiency and the downregulation of the mitochondria−nucleus signaling transduction caused by mitochondrial dysfunction lead to a gradual decline of neuronal function. In the study, we apply the healthy mitochondria to replace the dysfunctional mitochondria in neuronal cells and suggest that the mitotherapy could reduce the defective mitochondria and Aβ protein by NAD^+^/SIRT1−mediated autophagy, meanwhile enhancing the ATP production, thereby improving the cognitive function of AD. The clarification of the mechanism will be beneficial to facilitate the widespread application of mitotherapy in clinical settings.

Mitochondrion are active and dynamic organelles that act as the hubs of cellular signaling and metabolic activity in almost all eukaryotic cells, supporting organ functions and body health [55]. Mitochondrion are the major place of ATP production through OXPHOS in neurons. The neuronal cells have a high dependence on OXPHOS because of the high demand for energy and limited glycolytic ability [56,57,58]. Thus, mitochondrial dysfunction can greatly affect neuronal function. Moreover, ATP levels are also closely related to autophagy. Following damage to the mitochondrial function, autophagy is weakened due to the energy deficiency, despite autophagy being reported to have the ability to increase the energy supply by breaking down proteins and organelles. Nevertheless, energy is the basic condition for the initiation of autophagy, since the fusion between autophagosome and lysosome requires sufficient energy [59]. Decreased autophagy caused by an abnormal mitochondrial metabolism will promote the formation and accumulation of Aβ, which can aggravate mitochondrial injury through the production of a large amount of ROS [60,61,62,63]. The vicious circle will finally lead to neuronal dysfunction and cognitive impairment in AD patients. Recovery of normal mitochondrial function would prevent neuronal injury. Here, we used mitotherapy to restore the mitochondrial function, and then enhance the ATP content and decrease the Aβ level, resulting in the improvement of the cognitive ability of AD model mice.

NAD^+^ is an important signaling molecule from mitochondria to the nucleus that is mainly produced by the mitochondrial ETC complex I. Mitochondrial respiratory chain complex I, known as reduced nicotinamide adenine dinucleotide: coenzyme Q reductase (NADH: CoQ reductase), is the largest component in the mitochondrial electron−transport chain. The complex I is responsible for transforming NADH to NAD^+^. The decrease in the enzyme activity of complex I, the first reaction of the mitochondrial respiratory chain, is the most common event during mitochondrial damage. Following the activity of complex I decreasing, NAD^+^ production reduces, and excessive NADH accumulates. Therefore, NAD^+^/NADH can act as a marker of mitochondrial function. Meanwhile, NAD^+^ can act as a cofactor to activate transcription factors. SIRT1, the target enzyme of NAD^+^, is a histone deacetylase mainly expressed in neurons and which plays an important role in the growth and repair of neurons, antioxidation, autophagy, mitochondrial metabolism, and other processes [64,65,66]. The activity of SIRT1 is strictly regulated by the mitochondrial NAD^+^/NADH ratio. In the AD brain, the NAD^+^ content decreased and correspondingly SIRT1 activity was lower than the healthy elder population [67]. In addition, SIRT1 activity in the cerebral cortex of AD patients is negatively correlated with the degree of cognitive impairment [68,69,70,71]. The mice with the SIRT1 gene knockout exhibit poor spatial−learning ability and defective short−term and long−term memory [72]. After mitotherapy, the mitochondrial function is recovered, and then the NAD^+^/NADH ratio enhanced, leading to the activation of SIRT1 and its downstream signals.

One of the important cell events regulated by NAD^+^/SIRT1 is autophagy. Autophagy is a highly regulated mechanism of cells that degrade useless or dysfunctional components via the lysosomal pathway [73]. In addition, autophagy controls the mitochondrial quality during physiological conditions. However, the brains of AD patients and animal models show a significant autophagy defect, and a recent study reports that the autophagy disorder appears before Aβ deposition in AD patients [74,75,76,77]. Then, the misfolded Aβ protein and damaged mitochondria are unable to be removed due to the autophagy defect. Studies have shown that the impairment of the autophagy function can cause neurodegenerative diseases in humans, indicating that autophagy may play a crucially important role in the occurrence and progression of AD [78,79,80]. In the study, autophagy in the neuronal cells is activated by the NAD^+^/SIRT signal, evidenced by autophagy−related proteins, including FOXO3, BNIP3, and LC3, is upregulated, and several autophagosomes are observed under the TEM. Following the enhancement of the autophagy ability, the Aβ protein and damaged mitochondria are eliminated; then, the ROS induced by Aβ and the mitochondria decrease. Also, NAD^+^/SIRT1 can drive the BDNF protein expression and ERK phosphorylation, which will assist in repairing neuronal cells.

Currently, mitotherapy has attracted widespread attention in treating diseases related to mitochondrial dysfunction. Its effectiveness and safety have been indicated in clinical patients who suffer from myocardial ischemia and rare diseases. However, the molecular mechanism remains unclear. Here, we applied mitotherapy to improve the cognitive ability of AD and suggested that NAD^+^/SIRT1−mediated autophagy would be an important mechanism of the mitotherapy, in addition to the increased energy generation of the mitochondria themselves. The study suggests that mitotherapy would be a potential approach for AD treatment.

## Data Availability

The authors confirm that the data supporting the findings of this study are available within the article. Raw data are available from the corresponding author upon reasonable request.

## Abbreviations

AD, Alzheimer’s disease; Aβ, amyloid−β; BSA, bovine serum albumin; BCA, bicinchoninic acid; BDNF, brain−derived neurotrophic factor; CCCP, carbonyl cyanide m−chlorophenylhydrazone; DEGs, differential expression genes; ERK, extracellular−regulated protein kinases; ETC, electron-transport chain; GSH, reduced glutathione; GSSG, oxidized glutathione; GSH−Px, glutathione peroxidase; ICDHm, isocitrate dehydrogenase; MMP, mitochondrial membrane potential; MDA, malondialdehyde; MDC, monodansylcadaverine; MITOTHERAPY, transplantation therapy of healthy mitochondria; mtDNA, mitochondrial DNA; NAM, nicotinamide; OXPHOS, oxidative phosphorylation; PDH, pyruvate dehydrogenase; ROS, radical oxygen species; SOD, superoxide dismutase; SDH, succinate dehydrogenase; SIRT1, sirtuin 1; SD, standard deviation; T−AOC, total antioxidant capability; TEM, transmission electron microscopy; TCA, tricarboxylic acid; α−KGDH, α−ketoglutarate dehydrogenase.
